# Application of Chitosan-Based Hydrogel in Promoting Wound Healing: A Review

**DOI:** 10.3390/polym16030344

**Published:** 2024-01-26

**Authors:** Xueyan Che, Ting Zhao, Jing Hu, Kaicheng Yang, Nan Ma, Anning Li, Qi Sun, Chuanbo Ding, Qiteng Ding

**Affiliations:** 1 College of Traditional Chinese Medicine, Jilin Agriculture Science and Technology University, Jilin City 132101, China; cxy17376253128@163.com (X.C.); lyguiwandingding@163.com (T.Z.); 17657368028@163.com (J.H.); 15633806068@163.com (K.Y.); 18325968349@163.com (N.M.); 2 Jilin Aodong Yanbian Pharmaceutical Co., Ltd., Dunhua 133000, China; jladcpxstgb@163.com; 3 Jilin Zhengrong Pharmaceutical Development Co., Ltd., Dunhua 133700, China; sunqi001428@163.com; 4 College of Traditional Chinese Medicine, Jilin Agricultural University, Changchun 130118, China

**Keywords:** chitosan, hydrogel, wound healing

## Abstract

Chitosan is a linear polyelectrolyte with active hydroxyl and amino groups that can be made into chitosan-based hydrogels by different cross-linking methods. Chitosan-based hydrogels also have a three-dimensional network of hydrogels, which can accommodate a large number of aqueous solvents and biofluids. CS, as an ideal drug-carrying material, can effectively encapsulate and protect drugs and has the advantages of being nontoxic, biocompatible, and biodegradable. These advantages make it an ideal material for the preparation of functional hydrogels that can act as wound dressings for skin injuries. This review reports the role of chitosan-based hydrogels in promoting skin repair in the context of the mechanisms involved in skin injury repair. Chitosan-based hydrogels were found to promote skin repair at different process stages. Various functional chitosan-based hydrogels are also discussed.

## 1. Introduction

Hydrogels possess network structures in three dimensions (3D) composed of cross-linked polymer chains, enabling them to absorb substantial amounts of wound exudate. These hydrogels are distinguished by their elevated water content, soft composition, and porosity [1,2], exhibiting considerable potential as dressings for skin repair in recent years. Chitosan is the product of the chemical treatment of the deacetylation of chitin. It offers a variety of physiological functions, including biodegradability, biocompatibility, non-toxicity, bacteriostatic properties, anticancer potential, lipid-lowering capabilities, immune-enhancement effects, etc. Additionally, chitosan’s exceptional properties, such as its anticoagulant features and wound healing promotion, have led to its extensive employment in medical dressings [3,4]. The carboxyl and amino groups in chitosan can be utilized to create chitosan-based hydrogels with diverse properties. These hydrogels are considered excellent materials for wound dressing due to their biodegradable, biocompatible, and antimicrobial properties. Moreover, chitosan-based hydrogels hold great potential for clinical application in the treatment of skin wounds [5]. The properties of these hydrogels can be customized by incorporating different natural or synthetic polymers, resulting in functional chitosan-based hydrogels [6].

The process of skin repair Involves several stages: hemostasis, inflammation, proliferation, and tissue remodeling. During this process, various types of cells, such as epithelial cells, immune cells, endothelial cells, and fibroblasts, work together. Additionally, skin repair is influenced by environmental factors, but it is important because the skin serves as the body’s first line of defense against UV rays, microbial invasion, and other forms of aggression. As a result, prompt repair after a skin injury is of utmost importance [7].

Conventional wound dressings are currently unable to effectively address the requirements for treating chronic wound healing. However, novel smart chitosan-based hydrogel scaffolds have emerged as a potential solution. These hydrogels can be infused with active factors or drugs to not only inhibit inflammatory responses, oxidative stress, and microbial proliferation in chronic wounds but also promote the proliferation of skin cells. As a result, this review aims to summarize the recent applications of chitosan-based hydrogels in wound repair and explore the utilization of different drugs within these hydrogels. The findings of this review can serve as a valuable theoretical foundation for the future development and utilization of chitosan-based hydrogels.

## 2. Methods for Preparing Chitosan-Based Hydrogel

Chitosan is widely distributed in nature and is a renewable resource. People can extract chitosan from the shells of marine arthropods such as shrimps and crabs, mollusks and insects, and the cell walls of higher plants, and then prepare it by deacetylation [8]. Chitosan can be chemically and physically cross-linked to prepare a variety of chitosan-based hydrogels for wound repair (Table 1).

The main methods of physical cross-linking can be categorized into three ways. The first method involves electrostatic interaction, where the interaction between anionic molecules and the amino groups of chitosan leads to gelation. The second method is metal-ion coordination, which synthesizes gels through intermolecular coordination bonds, resulting in more stable hydrogels [14]. Yang J et al. utilized the α-hydroxyl in situ photoreduction and semi-soluble sol-gel transition (SD-A-SGT) method of silver ions and CMCTS to construct a multifunctional chitosan/carboxymethyl chitosan/silver nanoparticles (CTS/CMCTS/AgNPs) polyelectrolyte composite physical cross-linking hydrogel. The X-ray diffraction (XRD), transmission electron microscopy (TEM), and IR characterization revealed the highly uniform structure and good mechanical strength of the composite hydrogel. Additionally, the incorporation of AgNPs provided the hydrogel with excellent antimicrobial activity while also exhibiting good self-healing ability [10]. The third method involves hydrophobic interaction, where chitosan can undergo gelation through hydrophobic interactions. This biopolymer system allows for advantageous avoidance of potential side effects associated with in situ polymerization, such as monomer or initiator toxicity [15,16].

There are two main methods of chemical cross-linking. The first method is initiator-initiated cross-linking, where an initiator substance causes the monomer to undergo polymerization. This process allows chitosan and its derivative molecules to form a reticulation structure through covalent bonding, enhancing the material’s strength, elasticity, and other properties [17]. For example, Park et al. utilized ethylene glycol as a cross-linking agent and the blowing method to prepare a chitosan-based super porous hydrogel [18]. Another approach involves exposing chitosan matrices containing photoinitiators to high-energy UV radiation, which triggers interactions between molecular chains and promotes the formation of hydrogel structures. Drabczyk et al. synthesized chitosan hydrogels containing Aloe vera by UV radiation using a cross-linking agent (polyethylene glycol diacrylate), a photoinitiator (2-hydroxy-2-methylpropiophenone), chitosan, and Aloe vera juice (99.5%) [12]. The second method is the radiation cross-linking method, which utilizes radiation sources and substances to generate activated atoms and molecules, leading to cross-linking between substances. Fan et al. prepared chitosan (CS)/gelatin (Gel)/poly(vinyl alcohol) (PVA) hydrogels using the γ-irradiation method for wound dressing application. The physical properties and coagulation activity of these hydrogels were tested and yielded positive results. Additionally, the hydrogels exhibited good pH sensitivity, swelling ability, and water evaporation rate [19].

Physically cross-linked hydrogels are commonly made using biologically safe polymers as a matrix to avoid any potential adverse chemical reactions in the human body. However, these hydrogels often suffer from poor stability, weak tissue adhesion, and inadequate mechanical properties. To address these limitations, the incorporation of additional polymers is typically necessary during the preparation of physically cross-linked hydrogels. On the other hand, chemical hydrogels, which are formed through chemical bonding, necessitate comprehensive human hazard testing to ensure their biosafety. Furthermore, the production of chemically cross-linked hydrogels requires specialized techniques and specific conditions, which can be costly and have environmental implications. However, the advantage of chemically cross-linked hydrogels lies in their ability to be tailored for wound repair based on the specific wound type. Therefore, when developing chitosan-based hydrogels for wound repair, it is crucial to carefully consider both the wound characteristics and the biosafety of the hydrogel. This consideration is essential in selecting suitable hydrogel matrices and cross-linking methods.

## 3. Chitosan-Based Hydrogel for Skin Injury Repair

Skin wound healing can be further categorized into acute and chronic wounds, depending on the healing time. Acute wounds are usually caused by sudden abrasions, scratches, puncture wounds, etc. These wounds usually heal in 2–7 days for superficial wounds and in 2–4 weeks for deeper wounds. Even in the case of wounds with a small amount of bleeding and a small area, patients can treat them at home by themselves. The healing time of chronic wounds is usually more than four weeks [20], and the main common chronic wounds are burns [21], diabetic wounds [22,23,24], and decubitus ulcers [25,26]. Skin wounds are often accompanied by painful bleeding, and the long healing time means that they can cause prolonged suffering for the patient. Skin wound healing can be accelerated by the use of appropriate wound dressings during this process. The process of wound healing is complex and dynamic, involving various stages such as hemostasis, inflammation, proliferation, and tissue remodeling.

**Figure 1:**
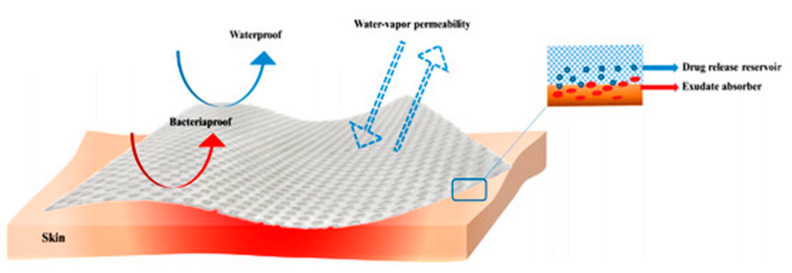
Ideal wound dressing [28], with permission from MDPI.

Ideal wound dressings are flexible, stable, biodegradable, and biocompatible, with the ability to maintain wound moistness, hemostasis, and the adsorption of exudate (refer to Figure 1), and chitosan-based hydrogels meet most of the conditions. Additionally, diverse variants of wound dressings composed of chitosan-based hydrogel possess the potential to enhance the healing process of wounds at varying time intervals and mitigate any detrimental factors impeding wound healing [27]. These formulations predominantly exert their functions during the initial three stages of wound healing. Primarily, they facilitate the cessation of bleeding by stimulating the aggregation of platelets and red blood cells while simultaneously impeding fibrinolysis during the hemostatic phase. Subsequently, they aid in the elimination of bacteria from the wound during the inflammatory phase. Lastly, they expedite the proliferation of skin cells by promoting the growth of granulation tissue, known as the proliferative phase. Consequently, the wound proceeds to recover, and the skin undergoes remodeling to ultimately accomplish the healing process.

### 3.1. Intelligent Chitosan-Based Hydrogel

Chitosan as a carrier can be physically and chemically modified to prepare multifunctional chitosan hydrogels to form smart hydrogels for releasing restorative components. Smart hydrogels produce a sol-gel phase transition while subjected to environmental stimuli such as pH, temperature, and light. Because of this property, smart chitosan-based hydrogels have also become a focus of research in the past few years. Smart chitosan-based hydrogels are usually heat-sensitive hydrogels, photosensitive hydrogels, and pH-sensitive hydrogels [29,30], as presented in Table 2.

Thermosensitive hydrogels are a type of hydrogels that exhibit temperature-sensitive behavior. These hydrogels remain in a sol state at low temperatures but undergo a phase change to a gel state when exposed to body temperature. This unique property makes them suitable for use as an alternative to conventional wound dressings. Chitosan temperature-sensitive hydrogel can be produced by incorporating specific materials such as β-glycerol phosphate, HPMC, and poloxamer [40]. Odinokov et al. synthesized pH- and thermosensitive hydrogels by cross-linking chitosan with Ter phthaloyl diazide [41]. Bhattarai et al. developed a hydrogel using a chitosan solution neutralized with a polyol counter-ionic single-head salt. This hydrogel remains in a liquid state at low temperatures and forms a gel at body temperature. These thermosensitive chitosan-based hydrogels show promise as therapeutic drug delivery systems for promoting skin tissue repair and regeneration. They achieve this through the controlled and sustained release of loaded drugs. Additionally, the injectable nature of these hydrogels allows for better adaptation to various irregular skin wounds. If used as a new type of dressing, they can closely conform to wounds with greater flexibility compared to traditional wound dressings [42].

The photosensitizer can be crosslinked with chitosan hydrogel to prepare a photoresponsive intelligent hydrogel, thereby expanding its application range. This hydrogel shows potential in drug delivery and can effectively inhibit bacterial growth through repeated photodynamic stimulation [43,44,45,46,47]. The combination of the photothermal effect and drug release, with the control of spatial and temporal factors, provides a synergistic therapy. This approach effectively prevents the disadvantages associated with separate treatment methods. The outstanding sterilizing effect of this therapy was demonstrated through in vitro antimicrobial assays and experiments on mouse wounds infected with *S. aureus*. Moreover, the hydrogel used in this treatment exhibits strong antioxidant properties. These properties are instrumental in eliminating the inflammatory response caused by the bacteria’s remnants at the site of infection. Consequently, this prevents any further harm to the tissue of the wound and facilitates its healing process [48].

The special feature of pH-sensitive hydrogels is their ability to change dimensions based on the pH of the environment [49]. During wound healing, the pH of the injured area undergoes dynamic changes. Normal skin typically has a pH below 5 [50]. However, when the skin surface is damaged, the underlying tissue with a pH of 7.4 becomes exposed. Chitosan, which has a pH of approximately 6.5, is responsive to these changes in environmental pH. In the early stages of wound healing, chitosan hydrogels expand in the acidic environment, which can enhance cellular infiltration, proliferation, and oxygen permeation. This hydrogel has potential applications in releasing anti-inflammatory agents during the initial wound-healing phase. Such release would reduce inflammation during the inflammatory phase and prevent excessive fibroblast growth during the proliferative phase [51].

Multiple studies have demonstrated that intelligent chitosan-based hydrogels exhibit superior adaptability to meet specific wound management requirements. These hydrogels offer promising solutions for treating special wounds and effectively leveraging the benefits of chitosan-based hydrogels in wound repair. However, it is important to note that these hydrogel products are still in the research phase, and further enhancements are required to improve their mechanical properties. To fully utilize them for wound treatment, extensive and thorough research is necessary.

### 3.2. Self-Healing Chitosan-Based Hydrogel

It is widely recognized that hydrogels can be injected to conform to different wound shapes. Injectable hydrogel wound dressings effectively fill and adhere to the wound site, protecting external factors. However, injectable hydrogels with weak elasticity may experience deformations or damage when exposed to mechanical forces after application. These damages can reduce the lifespan of the hydrogel, increase the risk of infections, and trigger inflammatory reactions [52,53,54]. The development of a hydrogel material with self-repairing ability has the potential to extend the lifespan of the hydrogel and minimize the occurrence of wound infections. In their study, Chen et al. successfully created a self-healing hydrogel using dynamic covalent bonding. This hydrogel exhibited remarkable self-healing properties, as it was able to regenerate a complete hydrogel structure even after being injected or fragmented due to injury [55]. Li et al. developed a biobased hydrogel by combining quaternized chitosan with bis-formaldehyde bacterial cellulose. The hydrogel exhibited rapid self-healing and injectable behavior, which was attributed to dynamic Schiff base interactions. Furthermore, it demonstrated remarkable antibacterial activity against both *Escherichia coli* (Gram negative) and Staphylococcus aureus (Gram positive). The hydrogels also showed suitable compression properties and an optimal water retention capacity [56].

Self-healing water gel offers self-healing capabilities for hydrogels due to the chemical bond linkage within the hydrogel matrix. With its unique properties, it is well-suited for wound dressings, effectively preventing damage to the hydrogel during daily activities. Furthermore, it can prolong the lifespan of the hydrogel, thereby reducing the discomfort experienced by patients during dressing changes and minimizing resource wastage.

### 3.3. Drug-Loaded Chitosan-Based Hydrogels

Chitosan-based hydrogels have emerged as an ideal material for the development of new-generation dressings, owing to their exceptional biocompatibility and wetting ability. Extensive research has demonstrated their efficacy in promoting the healing of acute wounds. However, the healing methods for chronic wounds differ. The selection of wound dressings for chronic wounds is contingent upon the specific conditions of the wound. Consequently, the treatment of chronic wounds necessitates specialized wound dressings. The limited effectiveness of chitosan-based hydrogels in promoting the healing of chronic wounds hampers their widespread application as wound dressings. To overcome this deficiency, researchers have explored the use of therapeutic drugs in conjunction with chitosan-based hydrogels to enhance their efficacy.

Drug loading in hydrogel scaffolds is a crucial strategy for enhancing the skin repair ability of wound dressings. By utilizing the pharmacological activity of drugs, they can be effectively employed for the treatment of chronic wounds through controlled release within hydrogel scaffolds. However, further experimental data are required to ascertain the biocompatibility of the loaded drug dosage.

#### 3.3.1. Loading Metal Ion Chitosan Hydrogel

From a clinical perspective, the presence of infectious bacteria can result in the suppuration of wounds, thereby prolonging the healing process [57]. This can further lead to the formation of wounds that are difficult to treat. In severe cases, it may even result in sepsis, posing a significant threat to life and overall well-being [58]. Consequently, the utilization of wound dressings with antibacterial and antimicrobial properties becomes crucial. Hydrogel dressings with antimicrobial capabilities can impede the growth of detrimental microorganisms, safeguard against microbial infections, and effectively minimize the risk of wound infection in patients. Extensive research has consistently demonstrated the remarkable bactericidal activity possessed by a variety of nanoparticles, including silver [59], copper [60,61], gold [62], and platinum [63,64]. The incorporation of these particles can significantly improve the antimicrobial activity of hydrogels. For example, Nešović et al. added silver nanoparticles (AgNPs), which are a broad-spectrum antimicrobial agent, to a hydrogel. The hydrogel showed strong antimicrobial activity in subsequent experiments [65].

#### 3.3.2. Chitosan-Based Hydrogel Loaded with Flavonoids

In recent years, flavonoids have garnered increased attention due to their significant antioxidant effects and their additional anti-inflammatory and antimicrobial properties. As a result, scholars are actively conducting in-depth research on flavonoids to develop new wound dressings. Apigenin, also known as apigenin and psoralen, is a flavonoid compound that is widely distributed in nature. It is primarily found in plants of the Rafflesiaceae, Verbenaceae, and Curculionaceae families, as well as in various vegetables and fruits in temperate zones. Celery, in particular, has a high content of apigenin. Numerous studies conducted both domestically and internationally have demonstrated the anti-tumor, cardiovascular, and cerebrovascular protective, antiviral, antibacterial, and other biological activities of apigenin. Building upon these properties, Shukla et al. proposed the development of a new wound dressing using a chitosan-based hydrogel loaded with apigenin. This dressing offers unique benefits such as biocompatibility, biodegradability, moisturization, and antioxidant efficacy, all of which effectively promote the healing of diabetic wounds [66].

Dihydroquercetin, also known as paclitaxel, is a potent flavonoid antioxidant. It is commonly found in onion, silymarin, French marine bark, and Douglas fir bark [67,68]. Ding et al. fabricated hydrogels sensitive to temperature by incorporating the bioactive compound taxifolin (TAX) into Poloxamer 407, chitosan (CS), and hyaluronic acid (HA). The findings demonstrated that the interconnected hydrogels showcased enhanced resistance to temperature changes, antioxidative properties, controlled drug release, and ensured safety. Moreover, the combination of paclitaxel and hydrogels expedited the healing process of skin injuries in mice [69].

#### 3.3.3. Chitosan-Based Hydrogels Loaded with Phenolic Acids

Phenolic acids, also known as phenolic compounds, are a class of compounds that contain multiple phenolic hydroxyl groups on the same benzene ring. Some examples of phenolic acids include caffeic acid, chlorogenic acid, pentapic acid, gallic acid, protocatechuic acid, ferulic acid, and mesic acid. These compounds have a wide range of physiological activities, such as antioxidant properties, free radical scavenging, protection against ultraviolet radiation, bacteriostatic effects, and antiviral effects. Among these activities, antioxidant and bacteriostatic effects play an important role in promoting wound healing.

Protocatechuic acid (3,4-dihydroxybenzoic acid, PCA) is a simple phenolic acid that can be found in various edible plants. It possesses a variety of pharmacological activities, including anti-inflammatory, antioxidant, antihyperglycemic, antimicrobial, anticancer, anti-aging, antitumor, antiasthmatic, antiulcer, and anti-spasmodic properties [70]. Zhou et al. experimented with developing injectable hydrogels with antioxidant properties. They used protocatechuic acid (PA) and carboxymethyl chitosan (CCS), which are conjugated with oxidized hyaluronic acid (OHA). The resulting OHA/CCS-PA composite hydrogels demonstrated excellent chemical stability, physical characteristics, and remarkable antimicrobial efficacy, surpassing 99% against common pathogens. Furthermore, these hydrogels showed satisfactory antioxidant capabilities by scavenging more than 85% of excessive free radicals, ultimately reducing cellular damage caused by oxidative stress. In vitro testing revealed that the sustained release of phenolic hydroxyl elements contributed to the reduction in reactive oxygen species expression. Notably, an animal study confirmed that the OHA/CCS-PA hydrogel outperformed commercially available Tegaderm™ film and a control hydrogel containing physically mixed PA, as it significantly enhanced wound healing with a remarkable 86.29% increase in collagen content. This indicates that OHA/CCS-PA hydrogels hold promising potential for wound healing applications [71].

Gallic acid (GA, 3,4,5-trihydroxybenzoic acid) is a natural phenolic antioxidant that can be extracted from plants, especially green tea [72]. Sun et al. prepared chitosan-copper-gallic acid nanocomposites (CS-Cu-GA NCs) with bifunctional nano-enzymatic properties (oxidative and peroxidase-like functions) using a combination of ionic cross-linking, in-situ reduction, and microwave-assisted methods. CS-Cu-GA NCs integrated the inherent antimicrobial properties of chitosan, Cu NPs, and Cu. Animal experiments showed that the antimicrobial dressings doped with CS-Cu-GA NCs could effectively promote the healing of Staphylococcus aureus-infected wounds with no damage to normal tissues. Additionally, the antimicrobial dressing was formulated into a bandage possessing exceptional water-swelling antimicrobial characteristics. Furthermore, it was securely affixed to medical adhesive tape in order to fabricate a portable antimicrobial commodity that can be administered to the human skin’s surface. This innovation showcases remarkable waterproof capabilities, thereby introducing novel perspectives regarding the development of antimicrobial products for clinical wound healing [73].

The aforementioned studies suggest that phenolic acid analogs have shown promising results in enhancing the antioxidant and antibacterial properties of chitosan-based hydrogels, thereby facilitating wound healing. These findings indicate that further research on the utilization of phenolic acid analogs in chitosan-based hydrogels can lead to the development of more efficient wound dressings.

#### 3.3.4. Chitosan-Based Hydrogel Carrying Plant Essential Oil

Plant essential oils are versatile substances that are extracted from various parts of plants, such as flowers, seeds, leaves, fruits, and roots. These oils are characterized by their hydrophobic, aromatic, and volatile nature, and they find widespread use in the food industry, perfumery, and aromatherapy. Notably, essential oils contain numerous bioactive components that possess antibacterial, antifungal, antiviral, insecticidal, and antioxidant properties, thereby making them highly suitable for diverse medical applications. In recent years, there has been research and application of essential oils to promote skin wound healing. Essential oils can effectively inhibit the growth of bacteria. The loading of essential oils on chitosan-based hydrogels is currently more intensively studied. Wang et al. investigated the antimicrobial activity of several plant essential oils, namely grape seed, rose, bergamot, lemon, chamomile, lavender, tea tree, ginger, cumin, and eucalyptus. The results showed that among them, EEO (eucalyptus essential oil), GEO (ginger essential oil), and CEO (cumin essential oil) exhibited the highest antimicrobial activity. Furthermore, due to their volatile nature, these essential oils are continuously released during the interaction with the hydrogel matrix. It was observed that when the added EEO, GEO, or CEO phases were added, they diffused on the surface of the hydrogel network structure, roughening the hydrogel surface and promoting cell adhesion, which is more favorable for wound repair [74]. Muscimol (2-isopropyl-5-methylphenol), a natural phenolic monoterpene found in essential oils mainly from thyme, oregano, and cow daylily, is one of the natural compounds with therapeutic abilities. Its various therapeutic properties, such as antioxidant, anti-inflammatory, local anesthetic, anti-injury sensory, distemper acid, antiseptic, and especially antibacterial and antifungal, have brought it to the forefront of interest in wound repair applications. Koosehgol et al. prepared blended films of chitosan, poly(ethylene glycol fumarate), and muscimol by solvent casting method. The films with a certain concentration of muscimol exhibited improved water vapor permeability, water vapor absorption, equilibrium water absorption, air permeability, swelling behavior, and antimicrobial activity against both Gram-negative and Gram-positive bacteria [75].

These studies demonstrate that the use of a plant essential oil in combination with a chitosan-based hydrogel has a positive effect on wound repair. However, it is widely recognized that plant essential oils are volatile, and further research is needed to investigate the stability of essential oil volatilization on the antibacterial properties of the gel and its impact on wound repair activity.

#### 3.3.5. Chitosan-Based Hydrogel Carrying Polypeptide

Peptide is a kind of amphoteric compound that is dehydrated from amino acids and contains carboxyl and amino groups. These compounds are easy to absorb, require low energy consumption to produce, and exhibit high affinity, specificity, and low toxicity. Existing studies have shown that peptides have the potential to stimulate tissue repair and wound healing [76,77,78]. Using chitosan-based hydrogels to carry peptides, stable delivery at the wound is more conducive to the peptide’s ability to promote skin wound healing. Ouyang et al. loaded tilapia peptide on chitosan-based hydrogel to prepare a wound dressing and explored the effectiveness of the dressing in treating burn wounds. The results showed that the chitosan-based hydrogel loaded with the peptide not only had good antimicrobial activity against *Escherichia coli* (*E. coli*) and *Staphylococcus aureus* (*S. aureus*) but also excellent antimicrobial activity. It also promotes skin regeneration [79,80].

Collagen is an extracellular protein known for its triple-helical structure. Its exceptional biodegradability, biocompatibility, antioxidant properties, and minimal immunogenic response make it a favorable option for various restorative applications. Collagen peptide (COP), a functional ingredient derived from collagen hydrolysis, possesses a lower molecular weight and is readily absorbed by the body. It offers advantages for bone health, Achilles tendon strength, and skin rejuvenation. Furthermore, COP facilitates cell attachment and proliferation [81]. Hu et al. grafted collagen peptide (COP) molecules onto the amino group of carboxymethyl chitosan sulfate (CMCS) using microbial glutamine transferase (MTGase) as a catalyst to enhance the antioxidant ability of hydrogels. The degree of substitution (DS) of CMCS-COP could be controlled experimentally by adjusting the reaction conditions. The ability of each sample to scavenge and reduce tends to increase significantly with increasing concentration. Meanwhile, no relevant cytotoxicity of the copolymer was found in NIH-3T3 mouse fibroblasts. These results indicate the promising potential of CMCS-COP as a novel wound dressing [82]. It is not difficult to see the role of chitosan-based hydrogels loaded with animal peptides in promoting wound repair. However, the use of animal peptides still carries some risks. Several factors limit the application of collagen from animal tissues. For example, their quality and purity can affect performance; non-human protein composition can lead to immune responses in susceptible patients; and there is a risk of contamination with pathogenic substances.

Recombinant human collagen peptides (RHCs) are considered reliable, predictable, and chemically defined as non-allergenic alternative biomaterials. Furthermore, studies have demonstrated that these RHCs are non-cytotoxic and can be effectively utilized in tissue engineering. Deng et al. discovered that by combining RHCs with chitosan, a thermosensitive hydrogel with improved mechanical properties was created, addressing the limitations of traditional thermosensitive hydrogels. This modified hydrogel was found to be more suitable for cell encapsulation and wound repair. The test results of the RHC chitosan hydrogel revealed that cells cultured with the modified hydrogel exhibited excellent vitality, in contrast to the hydrogel without RHC contact. Additionally, when injected into second-degree burned rats, the RHC chitosan hydrogel promoted cell infiltration, angiogenesis, and wound healing [83]. From the above, it is easy to see that chitosan-based hydrogels loaded with peptides have great potential as novel dressings for skin repair in the future.

#### 3.3.6. Chitosan-Based Hydrogel Carrying Other Therapeutic Components

In addition to the above types, Chitosan-based hydrogels can also be loaded with other therapeutic ingredients, such as bacteriostatic agents, antibiotics, phages, etc., which can inhibit bacteria, reduce wound infection, and promote chronic wound healing.

Antibiotics are chemical substances produced by microorganisms, plants, and animals that have properties that can potentially combat pathogens or disrupt the growth of other cells. There are several ways in which antibiotics can work against bacteria, including inhibiting cell wall synthesis, increasing cell membrane permeability, disrupting protein synthesis, and preventing nucleic acid replication and transcription. These mechanisms are the primary means by which antibiotics can destroy bacteria.

Amiri and colleagues investigated the incorporation of ticlopidine into chitosan-PEO nanofibers to enhance their antibacterial activity by 1.5 to 2 times. The results demonstrated that the ticlopidine-loaded nanofibers exhibited no cytotoxic effects on human fibroblasts. Additionally, in vivo experiments conducted using a rat total wound model confirmed the safety and effectiveness of utilizing ticlopidine-loaded nanofibers, which greatly accelerated wound closure [84]. Moghaddam et al. conducted a study in which they synthesized two O-carboxymethyl chitosan hydrogels combined with caffeic acid using electron beam irradiation. The researchers investigated the release of doxycycline from the chitosan-based hydrogel and found that it followed a non-Fick diffusion mechanism and exhibited different behavior in different media. In vitro release tests demonstrated that the composite hydrogel released more doxycycline compared to other matrices. The cytotoxicity study confirmed that the hydrogel dressings were non-toxic. Additionally, the chitosan hydrogel loaded with doxycycline showed inhibitory effects on the growth of Staphylococcus aureus and *Escherichia coli*. Therefore, the synthesized hydrogel holds the potential for the development of new wound dressings with antibacterial properties [85]. The application of antibiotics is more common in treating wounds and promoting wound healing. The loading of appropriate doses of antibiotics on chitosan-based hydrogels can be effective in antimicrobial treatment, reducing the risk of wound infection and promoting wound healing.

Antibiotic resistance has emerged as a significant concern within the medical community, particularly in the context of chronic wound infections caused by antibiotic-resistant bacteria. This global health issue arises due to the ability of bacteria to form biofilms on wound surfaces, facilitating their continued growth. As a consequence, the healing process becomes challenging and, in severe cases, can even result in death. To address this pressing issue, Fasiku et al. devised a hydrogel that employs a chitosan-based carrier infused with the antibacterial agent hydrogen peroxide (HP) and antibacterial peptides (Ps). This hydrogel can be directly applied to wounds, effectively combating biofilm-related bacteria, biofilms, and wound infections. The study findings demonstrate the hydrogel’s inhibitory activity against methicillin-resistant Staphylococcus aureus (MRSA) bacteria [86]. Ilomuanya et al., on the other hand, developed an encapsulation of a mixture of Acinetobacter baumannii phage by chitosan microparticles in a hydrogel matrix for the treatment of multidrug-resistant chronic wound infections. In vivo results showed a significant reduction in wound size [87]. These two studies mentioned above, in response to the problem of antibiotic misuse, have opened up the idea of applying antibiotics in correspondence to the challenges of wound repair. It brings hope for safer and more efficient wound repair in the future.

## 4. Process of Skin Repair

**Figure 2:**
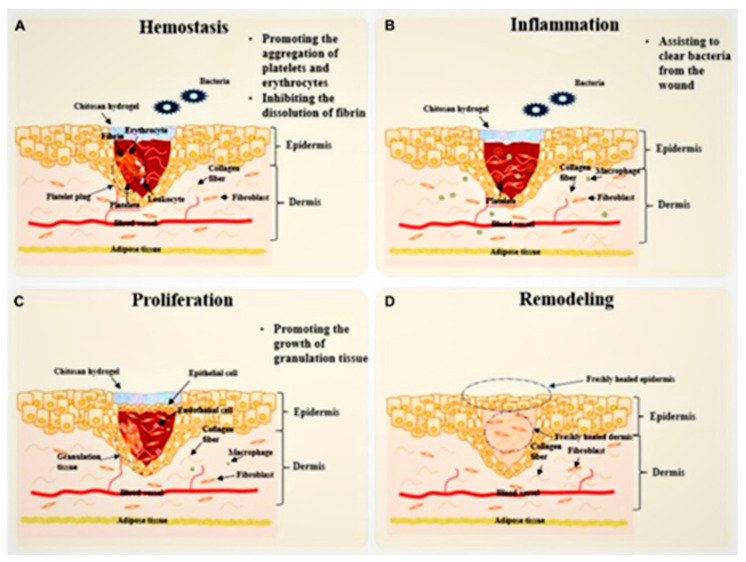
Four stages of skin repair [89], with permission from Frontiers. (**A**) Platelets, white blood cells, insoluble fibrin, and red blood cells combine to form a platelet thrombus, which effectively prevents bleeding during the hemostatic stage. Chitosan hydrogel plays a crucial role in hemostasis by enhancing the aggregation of platelets and red blood cells while inhibiting fibrin dissolution. (**B**) During the “inflammation” stage, chitosan hydrogel supports the activity of inflammatory cells, notably macrophages, aiding in the elimination of bacteria and necrotic tissue from the wound site. (**C**) The “proliferation” stage involves the proliferation and migration of epithelial cells to generate epithelial tissue, which acts as a protective layer for the wound. Chitosan hydrogel facilitates the growth of granulation tissue, effectively filling any tissue gaps that may exist. (**D**) The concluding stage encompasses reshaping, which signifies the completion of the entire skin repair process. Although chitosan-based hydrogels primarily exert their effects during the first three stages, their critical involvement in promoting healing is indisputable.

Skin wounds are common occurrences resulting from surgery, burns, chronic ulcers, and various traumatic injuries. Nevertheless, the process of wound healing is an intricate physiological phenomenon that is impacted by numerous elements [88]. Ordinarily, the complete process of wound healing encompasses four phases: hemostasis, inflammation, proliferation, and tissue remodeling (refer to Figure 2).

### 4.1. Hemostasis Stage

The creation of skin wounds inevitably leads to bleeding, and the process of repairing skin wounds begins with hemostasis. As soon as a skin wound starts bleeding, the organism’s spontaneous hemostatic response is triggered immediately. During the hemostatic phase, the coagulation system is activated after vasoconstriction and platelet aggregation. Fibrinogen is converted into insoluble fibrin, which forms a clot to stop the bleeding. However, the body’s spontaneous hemostatic function may not be sufficient for wound hemostasis in cases of large wound areas or other factors. Uncontrolled bleeding can result in various complications, including hypothermia, decreased blood pressure, bacterial infection, and even shock. If left untreated, it can lead to difficulties in wound healing and a significant increase in morbidity and mortality [90]. Bandages and gauze dressings are commonly used as traditional materials for wound hemostasis, relying on direct pressure to control bleeding. These materials have the advantages of being easily manufactured, cost-effective, and reusable. Nevertheless, their susceptibility to bacterial infection becomes evident in the presence of blood or tissue fluid [91]. In addition, dressing tears may lead to pain and increased wound healing time, plus they cannot accommodate irregular, deep, and narrow wound shapes. In addition to hemostasis by compression, there are other ways to stop bleeding. Topical hemostats, adhesives, and closures have been developed over the past few decades with good hemostatic results in surgical and emergency settings. Hemostatic agents enhance the blood-clotting cascade to achieve hemostasis, while adhesives hold various tissues and blood vessels together. However, both agents have limitations. For example, fibrin sealants based on fibrinogen and thrombin lack good adhesion properties and tend to shift during blood flushing. They may fail to stop bleeding and cause infection. Strong hemostatic adhesives, cyanoacrylates, have been developed to solve these problems, but they can cause allergic reactions [92]. In addition, cyanoacrylates rapidly generate heat during curing, and the degradation products may be toxic and have adverse effects on the human body [93]. Therefore, finding a safe, fast, and efficient hemostatic material is an urgent requirement.

**Figure 3:**
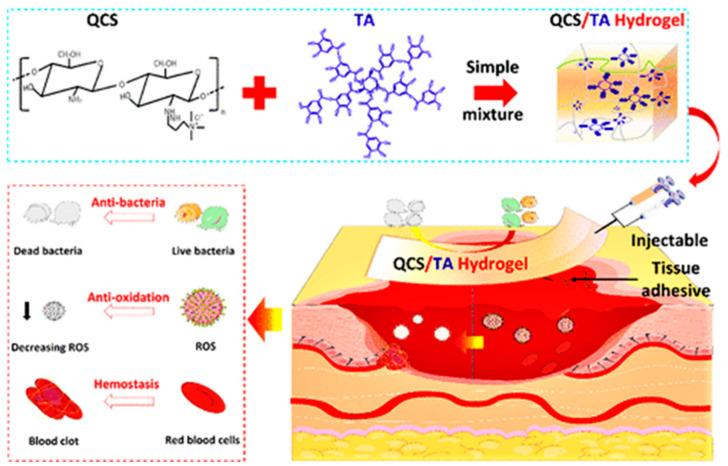
Chitosan-based hydrogels in the hemostatic phase [98]. Quaternary chitosan (QCS) Tannic acid (TA), with permission from ACS.

Chitosan has been found to have the ability to induce platelet and plasma protein aggregation, promoting coagulation and vasoconstriction at the site of injury [94,95]. It can also enhance the function of polymorphonuclear leukocytes, macrophages, and fibroblasts, leading to faster wound healing. Chitosan can be transformed into chitosan-based hydrogels that can be customized to fit irregular wound shapes (refer to Figure 3). Xia et al. developed a degradable chitosan-based hydrogel by cross-linking carboxymethyl chitosan with oxidized hyaluronic acid as the base material. This hydrogel exhibited significant hemostatic properties, as well as favorable rheology and cytocompatibility [96]. Similarly, Zhao et al. synthesized an antimicrobial antioxidant electroactive injectable hydrogel using quaternate chitosan-g-polyaniline (QCSP) and benzaldehyde-based functionalized poly (ethylene glycol)-copolymerization (sebacic acid glycerol) (PEGS-FA) as the main raw materials. The hemostatic properties of the hydrogel QCSP3/PEGS-FA were evaluated in a hemorrhagic liver mouse model. The application of the hydrogel at the bleeding site resulted In excellent hemostasis, with minimal blood stains on the filter paper, while the control group exhibited significant blood spots. Quantitative analysis of blood loss aligned with the macroscopic findings, showing a total blood loss of 214.7 ± 65.1 mg in the hydrogel adhesive group compared to 2025.9 ± 507.9 mg in the control group, indicating a significant difference (*p* < 0.01). The results showed that QCSP3/PEGSFA1.5 hydrogel has fast in situ gel properties, good adhesive properties, and excellent hemostatic properties, which makes it an effective anti-bleeding hydrogel barrier for practical applications [97].

The utilization of chitosan-based hydrogels during the hemostatic phase of wound healing has the potential to prevent life-threatening hemorrhage. Previous studies have demonstrated that chitosan, which carries a positive charge, interacts with activated platelets that carry a negative charge, thereby facilitating platelet adhesion and aggregation. Consequently, chitosan-based hydrogels can effectively contribute to the cessation of bleeding.

### 4.2. Inflammatory Stage

The second stage of skin repair is the inflammatory stage. Inflammation is an important response of the body’s immune system to ensure the survival of the body during infection and tissue damage. The inflammatory response is necessary to maintain normal tissue homeostasis. During the inflammatory phase, inflammatory cells remove bacteria and necrotic tissue. However, excessive and prolonged inflammatory reactions can be harmful instead of beneficial [99]. For the repair of damaged skin, a wound dressing with a good anti-inflammatory effect is required. Liu et al. developed alginate microspheres (Ms) loaded with basic fibroblast growth factor (bFGF) and incorporated them into carboxymethyl chitosan (CMCS)-poly (vinyl alcohol) (PVA) to create a composite chitosan-based hydrogel. The experimentally proven bFGF/Ms-CMCS-PVA composite hydrogel effectively affected inflammatory factors, inhibited inflammation, and successfully treated full-thickness skin burns on the backs of rats. Experiments have demonstrated that the bFGF/Ms-CMCS-PVA composite hydrogel has significant potential for rapidly restoring the structural and functional properties of damaged skin in burn patients [100]. Furthermore, Gull et al. also developed a hydrogel for the targeted release of diclofenac sodium with anti-inflammatory properties, using chitosan and poly (vinylidene beryllophthalide) as base polymers crosslinked with epichlorohydrin [101]. The prepared hydrogel exhibited good biodegradability, excellent antimicrobial properties, and promising cytotoxicity, as confirmed by in vitro studies. The drug release profile of the hydrogel showed 56.130% release of diclofenac sodium over 87 min, with a drug encapsulation efficiency of 84%.

The anti-inflammatory capacity of chitosan-based hydrogels alone may not be enough to inhibit the inflammatory response at the wound site and promote wound healing. However, researchers are exploring the incorporation of active ingredients with significant anti-inflammatory effects into chitosan-based hydrogel scaffolds. This area of research is considered a key focus in the development of tissue repair engineering scaffolds.

### 4.3. Proliferation Stage

The third stage of skin repair is proliferation, which involves the regeneration of tissues and the formation of granulation tissue. During this stage, epithelial cells proliferate and migrate to cover the wounds, while inflammatory cells, fibroblasts, and new capillaries form the granulation tissue. Research suggests that chitosan can accelerate skin wound repair by promoting the growth of inflammatory cells, fibroblasts, and capillaries. Chitosan stimulates the secretion of cytokines such as transforming growth factor-β (TGF-β), platelet-derived growth factor (PDGF), and interleukin-1 (IL-1) by macrophages. These cytokines play a crucial role in promoting migration, proliferation, collagen synthesis, and angiogenesis. Chitosan also increases the secretion of IL-8 by fibroblasts, further enhancing the inflammatory process and stimulating angiogenesis [102]. Chitosan hydrogels possess significant antibacterial properties and biocompatibility, making them highly promising in the fields of tissue engineering and regenerative medicine. However, the mechanical properties of pure chitosan hydrogels may be limited. In a study by Liu et al., a multifunctional chitosan hydrogel was developed using chitosan methacrylate (CTSMA) and vulcanized chitosan (CTSSH). The hydrogel was crosslinked through both free radical polymerization and mercaptan ene reaction. Comparatively, CTSMA/CTSSH (CMS) hydrogels demonstrated superior tissue adhesion and mechanical properties when compared to pure CTSMA hydrogels. Furthermore, this chitosan-based hydrogel showed potential in wound healing through its ability to promote angiogenesis, dermal repair, and epidermal regeneration [103].

### 4.4. Remodeling Phase

In the final remodeling phase, platelets in the blood clot secrete growth factors, including platelet-derived factor (PDGF), transforming growth factor (TGF)-α, and TGF-β, to promote wound healing. PDGF repairs connective tissue by attracting fibroblasts and promoting collagen deposition, thereby specifically promoting angiogenesis. Fresh epidermis and dermis will regenerate to complete the skin repair process [104]. Growth factors in skin wounds play a crucial role in promoting the healing process, specifically by regulating the proliferation, epithelialization, extracellular matrix remodeling, and angiogenesis of keratinocytes and fibroblasts. Chen et al. conducted a study to investigate the impact of chitosan hydrogel modified with SIKVAV (Ser Ile Lys Val Ala Val) peptide on skin wound healing. The experimental results demonstrate that the application of chitosan matrix hydrogel promotes the remodeling of skin wounds, thereby facilitating the wound healing process [105].

Based on the aforementioned research, chemical modification can be employed to enhance the limitations of chitosan-based hydrogels by combining them with other hydrogel matrices. Nevertheless, it is crucial to conduct comprehensive biocompatibility tests to ensure the safety of modified chitosan in hydrogel applications.

## 5. Conclusions and Prospects

In recent years, researchers have conducted studies on smart chitosan-based hydrogels. One notable example is the addition of silver and other metal nanoparticles with antimicrobial activity to chitosan-based pH-responsive hydrogels. This addition has resulted in hydrogels with high antimicrobial activity, which have been successfully used in experimental treatments for full-layer skin burns. These treatments have shown excellent therapeutic efficacy and effectively prevent wound infections. Additionally, there are various types of smart chitosan-based hydrogels, including thermosensitive, photosensitive, and pH-sensitive hydrogels. Thermosensitive hydrogels exhibit reversible sol-gel properties, while photosensitive hydrogels utilize light stimulation to enhance bacterial inhibition. pH-sensitive hydrogels respond to the different pH environments of wounds and promote wound healing. Another area of research focuses on self-repairing chitosan-based hydrogels, which have the potential to increase the lifespan of hydrogels. Furthermore, pharmaceutical hydrogels can be loaded with potent drugs to promote wound repair. These advancements represent significant progress in the field of chitosan-based hydrogels.

There are currently several types of chitosan-based hydrogel wound dressings available in the market. However, these products do not fully meet the requirements for clinical applications. While the extraction process of chitosan is well established, the technology for industrialized production of hydrogels is still in its early stages. Further research is needed to improve the preparation methods of hydrogels, enabling their stable use in industrial production. Collaboration between researchers and enterprises is crucial to promoting the application of chitosan-based hydrogels in wound repair. Additionally, there are some challenges that need to be addressed, such as the poor adhesion of chitosan-based hydrogels and the precise control of drug release from drug-carrying hydrogels. Biosafety concerns also need to be fully considered during the preparation of chitosan-based hydrogels for wound repair. In conclusion, chitosan-based hydrogels have great potential for wound repair applications. However, it is important to thoroughly evaluate the stability, safety, and reliability of the preparation method, and the feasibility of industrialized production of hydrogels during their development.

## Figures and Tables

**Table 1:** Preparation of chitosan-based hydrogels.

Cross-Linking Type	Method	Mechanism	Refs.
Physical cross-linking	Electrostatic interaction	Electrostatic interactions occur through an interaction between anionic molecules and the amino groups of chitosan to gelate them.	[9]
	Metal ion coordination	Metal ion coordination is used to synthesize gels through intermolecular coordination bonds, forming more stable hydrogels.	[10]
	Hydrophobic interaction	Chitosan can undergo gelation through hydrophobic interactions, and this interacting biopolymer system can advantageously avoid the potential side effects of in situ polymerization associated with monomer or initiator toxicity.	[11]
Chemical cross-linking	Cross-linking agent initiated cross-linking method	Initiators are substances that can cause monomers to undergo polymerization, which can cause chitosan and its derivative molecules to combine through covalent bonds to form a reticulated structure and improve the strength, elasticity, and other properties of the material.	[12]
Radiation cross-linking		The radiation cross-linking method uses the action of a radiation source with a substance to ionize and excite the generation of activated atoms and molecules, causing cross-linking between the substances.	[13]

**Table 2:** Smart chitosan-based hydrogels.

Type	Added Ingredients	Action Mechanism	Refs.
pH - sensitive	Red cabbage extract (RCE)/ chitosan (cs)/Methylenebisacrylamide (MBAA)	The colorimetric properties of RCE-loaded chitosan hydrogels show that RCE undergoes visual color changes in both acidic and alkaline media. Monitoring wound pH changes can protect, heal, and indicate the healing process	[31]
	Polyacrylamide-quaternary ammonium/chitosan-carbon quantum dots (CQDs) phenol red hydrogel	Hybridization of CQD and pH indicator (phenol red) with the hydrogel resulted in a highly responsive, reversible, and accurate indication of pH variability to reflect dynamic wound states in both UV and visible backgrounds.	[32]
Heat - sensitive	Curcumin/Carboxymethyl/cellulose	Cur-micellar-loaded hydrogels promote tissue regenerative capacity with enhanced fibroblasts, keratin-forming cells, and collagen deposition to stimulate epidermal junctions. Interestingly, chitosan-CMC-g-PF127 injectable hydrogel exhibited rapid wound repair potential by increasing cell migration and proliferation at the site of injury and providing a continuous drug delivery platform to the hydrophobic fraction.	[33]
	Synthesis by free radical-mediated polymerization of tannic acid-assisted New-gel hydrogels.	The new gel has excellent chemical/physical properties and can effectively load and release drugs and maintain drug activity. At the same time, New-gel has excellent oxygen loading capacity, which provides significant practical therapeutic benefits for diabetic wound repair.	[34]
Photo - responsive	Prussian blue nanoparticles (PBNPs)/Glycidyl trimethylammonium chloride (GTAC)/ Glycidyl methacrylate (GMA)	The positively charged QC in the hydrogel can capture bacteria through electrostatic attraction, change the potential of the bacterial membrane, destroy the bacterial membrane, and ultimately reduce the activity of the bacteria or even kill them. At the same time, the heat therapy generated by PBNPs under near-infrared (NIR) light irradiation can effectively and quickly kill these weak bacteria at mild temperatures (<55 °C).	[35]
	Porphyrin photosensitizer/polylactic acid-glycolic acid (PLGA)/basic fibroblast growth factor (bFGF)/carboxymethyl chitosan (CMCS)	The hydrogel block helps with repeated photodynamic stimulation and inhibits bacterial growth, while the aFGF content promotes wound healing.	[36]
Magnetic field corresponding hydrogel	Magnetite precursor/chitosan	The direct remote control of drug release behavior using low-frequency alternating magnetic field (LAMF) also avoids potential adverse thermal effects.	[37]
Electric field responsive hydrogel	Chitosan (CS)/Hydroxyethyl cellulose oxide (OHEC)/Reduced graphene oxide (RGO)/Salicylide liposomes	CS/OHEC/rGO/asiaticoside liposome hydrogel was prepared by dispersing RGO and asiaticoside into the hydrogel. This hydrogel serves as a filler for hollow nerve conduits, leveraging the benefits of OHEC and CS to enhance the mechanical and degradation properties of CS. Moreover, the hydrogel incorporates conductive rGO, which facilitates electric stimulation and scar inhibition, thereby promoting peripheral nerve regeneration.	[38]
Plasma-activated hydrogel	Gallic acid-modified chitosan-based (CS-GA)	Through self-cross-linking reaction exposed to oxygen, CS-GA solution may become bioadhesive hydrogel with high biocompatibility and blood compatibility, which is helpful for wound healing and hemostasis.	[39]

## Data Availability

No data was used for the research described in the article.

## References

[1] Ho T.C., Chang C.C., Chan H.P., Chung T.W., Shu C.W., Chuang K.P., Duh T.H., Yang M.H., Tyan Y.C. (2022). Hydrogels: Properties and applications in biomedicine. Molecules.

[2] Brumberg V., Astrelina T., Malivanova T., Samoilov A. (2021). Modern wound dressings: Hydrogel dressings. Biomedicines.

[3] Ogawa K., Yui T., Okuyama K. (2004). Three d structures of chitosan. Int. J. Biol. Macromol..

[4] Hamedi H., Moradi S., Hudson S.M., Tonelli A.E. (2018). Chitosan based hydrogels and their applications for drug delivery in wound dressings: A review. Carbohydr. Polym..

[5] Desai N., Rana D., Salave S., Gupta R., Patel P., Karunakaran B., Sharma A., Giri J., Benival D., Kommineni N. (2023). Chitosan: A potential biopolymer in drug delivery and biomedical applications. Pharmaceutics.

[6] Pella M., Lima-Tenorio M.K., Tenorio-Neto E.T., Guilherme M.R., Muniz E.C., Rubira A.F. (2018). Chitosan-based hydrogels: From preparation to biomedical applications. Carbohydr. Polym..

[7] Broughton G.N., Janis J.E., Attinger C.E. (2006). Wound healing: An overview. Plast. Reconstr. Surg..

[8] Tian B., Hua S., Tian Y., Liu J. (2020). Chemical and physical chitosan hydrogels as prospective carriers for drug delivery: A review. J. Mat. Chem. B.

[9] Kim G.O., Kim N., Kim D.Y., Kwon J.S., Min B.H. (2012). An electrostatically crosslinked chitosan hydrogel as a drug carrier. Molecules.

[10] Yang J., Chen Y., Zhao L., Feng Z., Peng K., Wei A., Wang Y., Tong Z., Cheng B. (2020). Preparation of a chitosan/carboxymethyl chitosan/agnps polyelectrolyte composite physical hydrogel with self-healing ability, antibacterial properties, and good biosafety simultaneously, and its application as a wound dressing. Compos. Pt. B Eng..

[11] Mirzaei E.B., Ramazani A.S.A., Shafiee M., Danaei M. (2013). Studies on glutaraldehyde crosslinked chitosan hydrogel properties for drug delivery systems. Int. J. Polym. Mater. Polym. Biomat..

[12] Drabczyk A., Kudlacik-Kramarczyk S., Glab M., Kedzierska M., Jaromin A., Mierzwinski D., Tyliszczak B. (2020). Physicochemical investigations of chitosan-based hydrogels containing aloe vera designed for biomedical use. Materials.

[13] Nguyen N.T., Liu J.H. (2013). Fabrication and characterization of poly (vinyl alcohol)/chitosan hydrogel thin films via uv irradiation. Eur. Polym. J..

[14] Pita-Lopez M.L., Fletes-Vargas G., Espinosa-Andrews H., Rodriguez-Rodriguez R. (2021). Physically cross-linked chitosan-based hydrogels for tissue engineering applications: A state-of-the-art review. Eur. Polym. J..

[15] Schuetz Y.B., Gurny R., Jordan O. (2008). A novel thermoresponsive hydrogel based on chitosan. Eur. J. Pharm. Biopharm..

[16] Xu Y., Yuan S., Han J., Lin H., Zhang X. (2017). Design and fabrication of a chitosan hydrogel with gradient structures via a step-by-step cross-linking process. Carbohydr. Polym..

[17] Berger J., Reist M., Mayer J.M., Felt O., Peppas N.A., Gurny R. (2004). Structure and interactions in covalently and ionically crosslinked chitosan hydrogels for biomedical applications. Eur. J. Pharm. Biopharm..

[18] Park H., Park K., Kim D. (2006). Preparation and swelling behavior of chitosan-based superporous hydrogels for gastric retention application. J. Biomed. Mater. Res. Part A.

[19] Fan L., Yang H., Yang J., Peng M., Hu J. (2016). Preparation and characterization of chitosan/gelatin/pva hydrogel for wound dressings. Carbohydr. Polym..

[20] Siddiqui A.R., Bernstein J.M. (2010). Chronic wound infection: Facts and controversies. Clin. Dermatol..

[21] (2020). Burn injury. Nat. Rev. Dis. Primers.

[22] Falanga V. (2005). Wound healing and its impairment in the diabetic foot. Lancet.

[23] Peppa M., Stavroulakis P., Raptis S.A. (2009). Advanced glycoxidation products and impaired diabetic wound healing. Wound Repair Regen..

[24] Burgess J.L., Wyant W.A., Abdo A.B., Kirsner R.S., Jozic I. (2021). Diabetic wound-healing science. Medicina.

[25] Kottner J., Black J., Call E., Gefen A., Santamaria N. (2018). Microclimate: A critical review in the context of pressure ulcer prevention. Clin. Biomech..

[26] Vangilder C., Lachenbruch C., Algrim-Boyle C., Meyer S. (2017). The international pressure ulcer prevalence survey: 2006–2015: A 10-year pressure injury prevalence and demographic trend analysis by care setting. J. Wound Ostomy Cont. Nurs..

[27] Liu H., Wang C., Li C., Qin Y., Wang Z., Yang F., Li Z., Wang J. (2018). A functional chitosan-based hydrogel as a wound dressing and drug delivery system in the treatment of wound healing. RSC Adv..

[28] Negut I., Grumezescu V., Grumezescu A.M. (2018). Treatment strategies for infected wounds. Molecules.

[29] Mu M., Li X., Tong A., Guo G. (2019). Multi-functional chitosan-based smart hydrogels mediated biomedical application. Expert Opin. Drug Deliv..

[30] Taokaew S., Kaewkong W., Kriangkrai W. (2023). Recent development of functional chitosan-based hydrogels for pharmaceutical and biomedical applications. Gels.

[31] Arafa A.A., Nada A.A., Ibrahim A.Y., Sajkiewicz P., Zahran M.K., Hakeim O.A. (2021). Preparation and characterization of smart therapeutic ph-sensitive wound dressing from red cabbage extract and chitosan hydrogel. Int. J. Biol. Macromol..

[32] Zheng K., Tong Y., Zhang S., He R., Xiao L., Iqbal Z., Zhang Y., Gao J., Zhang L., Jiang L. (2021). Flexible bicolorimetric polyacrylamide/chitosan hydrogels for smart real—Time monitoring and promotion of wound healing. Adv. Funct. Mater..

[33] Shah S.A., Sohail M., Karperien M., Johnbosco C., Mahmood A., Kousar M. (2023). Chitosan and carboxymethyl cellulose-based 3D multifunctional bioactive hydrogels loaded with nano-curcumin for synergistic diabetic wound repair. Int. J. Biol. Macromol..

[34] Cai Y., Fu X., Zhou Y., Lei L., Wang J., Zeng W., Yang Z. (2023). A hydrogel system for drug loading toward the synergistic application of reductive/heat-sensitive drugs. J. Control. Release.

[35] Han D., Li Y., Liu X., Li B., Han Y., Zheng Y., Yeung K.W.K., Li C., Cui Z., Liang Y. (2020). Rapid bacteria trapping and killing of metal-organic frameworks strengthened photo-responsive hydrogel for rapid tissue repair of bacterial infected wounds. Chem. Eng. J..

[36] Mai B., Jia M., Liu S., Sheng Z., Li M., Gao Y., Wang X., Liu Q., Wang P. (2020). Smart hydrogel-based dvdms/bfgf nanohybrids for antibacterial phototherapy with multiple damaging sites and accelerated wound healing. ACS Appl. Mater. Interfaces.

[37] Wang Y., Li B., Xu F., Han Z., Wei D., Jia D., Zhou Y. (2018). Tough magnetic chitosan hydrogel nanocomposites for remotely stimulated drug release. Biomacromolecules.

[38] Zheng F., Li R., He Q., Koral K., Tao J., Fan L., Xiang R., Ma J., Wang N., Yin Y. (2020). The electrostimulation and scar inhibition effect of chitosan/oxidized hydroxyethyl cellulose/reduced graphene oxide/asiaticoside liposome based hydrogel on peripheral nerve regeneration in vitro. Mater. Sci. Eng. C.

[39] Sun C., Zeng X., Zheng S., Wang Y., Li Z., Zhang H., Nie L., Zhang Y., Zhao Y., Yang X. (2022). Bio-adhesive catechol-modified chitosan wound healing hydrogel dressings through glow discharge plasma technique. Chem. Eng. J..

[40] Blacklow S.O., Li J., Freedman B.R., Zeidi M., Chen C., Mooney D.J. (2019). Bioinspired mechanically active adhesive dressings to accelerate wound closure. Sci. Adv..

[41] Odinokov A.V., Dzhons D.Y., Budruev A.V., Mochalova A.E., Smirnova L.A. (2016). Chitosan modified with terephthaloyl diazide as a drug delivery system. Russ. Chem. Bull..

[42] Bhattarai N., Ramay H.R., Gunn J., Matsen F.A., Zhang M. (2005). Peg-grafted chitosan as an injectable thermosensitive hydrogel for sustained protein release. J. Control. Release.

[43] He M., Han B., Jiang Z., Yang Y., Peng Y., Liu W. (2017). Synthesis of a chitosan-based photo-sensitive hydrogel and its biocompatibility and biodegradability. Carbohydr. Polym..

[44] Chen X., Li H., Lam K.Y. (2020). A multiphysics model of photo-sensitive hydrogels in response to light-thermo-pH-salt coupled stimuli for biomedical applications. Bioelectrochemistry.

[45] Tomatsu I., Peng K., Kros A. (2011). Photoresponsive hydrogels for biomedical applications. Adv. Drug Deliv. Rev..

[46] Li L., Scheiger J.M., Levkin P.A. (2019). Design and applications of photoresponsive hydrogels. Adv. Mater..

[47] Liu J., Xiao Y., Wang X., Huang L., Chen Y., Bao C. (2019). Glucose-sensitive delivery of metronidazole by using a photo-crosslinked chitosan hydrogel film to inhibit porphyromonas gingivalis proliferation. Int. J. Biol. Macromol..

[48] Yang N., Zhu M., Xu G., Liu N., Yu C. (2020). A near-infrared light-responsive multifunctional nanocomposite hydrogel for efficient and synergistic antibacterial wound therapy and healing promotion. J. Mat. Chem. B.

[49] Yan H., Jin B. (2013). Equilibrium swelling of a polyampholytic pH-sensitive hydrogel. Eur. Phys. J. E.

[50] Lambers H., Piessens S., Bloem A., Pronk H., Finkel P. (2006). Natural skin surface pH is on average below 5, which is beneficial for its resident flora. Int. J. Cosmet. Sci..

[51] Wu J., Su Z.G., Ma G.H. (2006). A thermo- and pH-sensitive hydrogel composed of quaternized chitosan/glycerophosphate. Int. J. Pharm..

[52] Ren Z., Ke T., Ling Q., Zhao L., Gu H. (2021). Rapid self-healing and self-adhesive chitosan-based hydrogels by host-guest interaction and dynamic covalent bond as flexible sensor. Carbohydr. Polym..

[53] Ou Y., Tian M. (2021). Advances in multifunctional chitosan-based self-healing hydrogels for biomedical applications. J. Mat. Chem. B.

[54] Li L., Yan B., Yang J., Chen L., Zeng H. (2015). Novel mussel-inspired injectable self-healing hydrogel with anti-biofouling property. Adv. Mater..

[55] Chen M., Tian J., Liu Y., Cao H., Li R., Wang J., Wu J., Zhang Q. (2019). Dynamic covalent constructed self—Healing hydrogel for sequential delivery of antibacterial agent and growth factor in wound healing. Chem. Eng. J..

[56] Deng L., Wang B., Li W., Han Z., Chen S., Wang H. (2022). Bacterial cellulose reinforced chitosan-based hydrogel with highly efficient self-healing and enhanced antibacterial activity for wound healing. Int. J. Biol. Macromol..

[57] Yu Q., Yan Y., Huang J., Liang Q., Li J., Wang B., Ma B., Bianco A., Ge S., Shao J. (2023). A multifunctional chitosan-based hydrogel with self-healing, antibacterial, and immunomodulatory effects as wound dressing. Int. J. Biol. Macromol..

[58] Robson M.C. (1997). Wound infection. A failure of wound healing caused by an imbalance of bacteria. Surg. Clin. North Am..

[59] Wang C., Huang X., Deng W., Chang C., Hang R., Tang B. (2014). A nano-silver composite based on the ion-exchange response for the intelligent antibacterial applications. Mater. Sci. Eng. C-Mater. Biol. Appl..

[60] Chatterjee A.K., Chakraborty R., Basu T. (2014). Mechanism of antibacterial activity of copper nanoparticles. Nanotechnology.

[61] Tamayo L., Azocar M., Kogan M., Riveros A., Paez M. (2016). Copper-polymer nanocomposites: An excellent and cost-effective biocide for use on antibacterial surfaces. Mater. Sci. Eng. C-Mater. Biol. Appl..

[62] Ramamurthy C.H., Padma M., Samadanam I.D., Mareeswaran R., Suyavaran A., Kumar M.S., Premkumar K., Thirunavukkarasu C. (2013). The extra cellular synthesis of gold and silver nanoparticles and their free radical scavenging and antibacterial properties. Colloid Surf. B Biointerfaces.

[63] Odularu A.T., Ajibade P.A., Mbese J.Z., Oyedeji O.O. (2019). Developments in platinum-group metals as dual antibacterial and anticancer agents. J. Chem..

[64] Liu H., Du Y., Wang X., Sun L. (2004). Chitosan kills bacteria through cell membrane damage. Int. J. Food Microbiol..

[65] Nesovic K., Jankovic A., Radetic T., Vukasinovic-Sekulic M., Kojic V., Zivkovic L., Peric-Grujic A., Rhee K.Y., Miskovic-Stankovic V. (2019). Chitosan-based hydrogel wound dressings with electrochemically incorporated silver nanoparticles—In vitro study. Eur. Polym. J..

[66] Shukla R., Kashaw S.K., Jain A.P., Lodhi S. (2016). Fabrication of apigenin loaded gellan gum-chitosan hydrogels (ggch-hgs) for effective diabetic wound healing. Int. J. Biol. Macromol..

[67] Ding C., Zhao Y., Chen X., Zheng Y., Liu W., Liu X. (2021). Taxifolin, a novel food, attenuates acute alcohol-induced liver injury in mice through regulating the nf-κb-mediated inflammation and pi3k/akt signalling pathways. Pharm. Biol..

[68] Ding Q., Ding C., Liu X., Zheng Y., Zhao Y., Zhang S., Sun S., Peng Z., Liu W. (2023). Preparation of nanocomposite membranes loaded with taxifolin liposome and its mechanism of wound healing in diabetic mice. Int. J. Biol. Macromol..

[69] Ding C., Liu Z., Zhao T., Sun S., Liu X., Zhang J., Ma L., Yang M. (2023). A temperature-sensitive hydrogel loaded with taxifolin promotes skin repair by modulating mapk-mediated autophagic pathway. J. Mater. Sci..

[70] Khan A.K., Rashid R., Fatima N., Mahmood S., Mir S., Khan S., Jabeen N., Murtaza G. (2015). Pharmacological activities of protocatechuic acid. Acta Pol. Pharm..

[71] Zhou C., Xu R., Han X., Tong L., Xiong L., Liang J., Sun Y., Zhang X., Fan Y. (2023). Protocatechuic acid-mediated injectable antioxidant hydrogels facilitate wound healing. Compos. Pt. B Eng..

[72] Pasanphan W., Chirachanchai S. (2008). Conjugation of gallic acid onto chitosan: An approach for green and water-based antioxidant. Carbohydr. Polym..

[73] Sun X., Dong M., Guo Z., Zhang H., Wang J., Jia P., Bu T., Liu Y., Li L., Wang L. (2021). Multifunctional chitosan-copper-gallic acid based antibacterial nanocomposite wound dressing. Int. J. Biol. Macromol..

[74] Wang H., Liu Y., Cai K., Zhang B., Tang S., Zhang W., Liu W. (2021). Antibacterial polysaccharide-based hydrogel dressing containing plant essential oil for burn wound healing. Burn. Trauma.

[75] Koosehgol S., Ebrahimian-Hosseinabadi M., Alizadeh M., Zamanian A. (2017). Preparation and characterization of in situ chitosan/polyethylene glycol fumarate/thymol hydrogel as an effective wound dressing. Mater. Sci. Eng. C-Mater. Biol. Appl..

[76] Pickart L. (2008). The human tri-peptide ghk and tissue remodeling. J. Biomater. Sci.-Polym. Ed..

[77] Pickart L., Vasquez-Soltero J.M., Margolina A. (2015). Ghk peptide as a natural modulator of multiple cellular pathways in skin regeneration. Biomed. Res. Int..

[78] Wang S., Feng C., Yin S., Feng Z., Tang J., Liu N., Yang F., Yang X., Wang Y. (2021). A novel peptide from the skin of amphibian rana limnocharis with potency to promote skin wound repair. Nat. Prod. Res..

[79] Ouyang Q.Q., Hu Z., Lin Z.P., Quan W.Y., Deng Y.F., Li S.D., Li P.W., Chen Y. (2018). Chitosan hydrogel in combination with marine peptides from tilapia for burns healing. Int. J. Biol. Macromol..

[80] Qianqian O., Songzhi K., Yongmei H., Xianghong J., Sidong L., Puwang L., Hui L. (2021). Preparation of nano-hydroxyapatite/chitosan/tilapia skin peptides hydrogels and its burn wound treatment. Int. J. Biol. Macromol..

[81] Xiao Y., Ge H., Zou S., Wen H., Li Y., Fan L., Xiao L. (2017). Enzymatic synthesis of n-succinyl chitosan-collagen peptide copolymer and its characterization. Carbohydr. Polym..

[82] Hu W., Liu M., Yang X., Zhang C., Zhou H., Xie W., Fan L., Nie M. (2019). Modification of chitosan grafted with collagen peptide by enzyme crosslinking. Carbohydr. Polym..

[83] Deng A., Yang Y., Du S., Yang X., Pang S., Wang X., Yang S. (2021). Preparation of a recombinant collagen-peptide (rhc)-conjugated chitosan thermosensitive hydrogel for wound healing. Mater. Sci. Eng. C-Mater. Biol. Appl..

[84] Amiri N., Ajami S., Shahroodi A., Jannatabadi N., Amiri D.S., Fazly B.B., Pishavar E., Kalalinia F., Movaffagh J. (2020). Teicoplanin-loaded chitosan-peo nanofibers for local antibiotic delivery and wound healing. Int. J. Biol. Macromol..

[85] Hafezi M.R., Dadfarnia S., Shabani A., Amraei R., Hafezi M.Z. (2020). Doxycycline drug delivery using hydrogels of o-carboxymethyl chitosan conjugated with caffeic acid and its composite with polyacrylamide synthesized by electron beam irradiation. Int. J. Biol. Macromol..

[86] Fasiku V.O., Omolo C.A., Devnarain N., Ibrahim U.H., Rambharose S., Faya M., Mocktar C., Singh S.D., Govender T. (2021). Chitosan-based hydrogel for the dual delivery of antimicrobial agents against bacterial methicillin-resistant staphylococcus aureus biofilm-infected wounds. ACS Omega.

[87] Ilomuanya M.O., Enwuru N.V., Adenokun E., Fatunmbi A., Adeluola A., Igwilo C.I. (2022). Chitosan-based microparticle encapsulated acinetobacter baumannii phage cocktail in hydrogel matrix for the management of multidrug resistant chronic wound infection. Turk. J. Pharm. Sci..

[88] Guo S., Dipietro L.A. (2010). Factors affecting wound healing. J. Dent. Res..

[89] Feng P., Luo Y., Ke C., Qiu H., Wang W., Zhu Y., Hou R., Xu L., Wu S. (2021). Chitosan-based functional materials for skin wound repair: Mechanisms and applications. Front. Bioeng. Biotechnol..

[90] Hoshi R., Murata S., Matsuo R., Myronovych A., Hashimoto I., Ikeda H., Ohkohchi N. (2007). Freeze-dried platelets promote hepatocyte proliferation in mice. Cryobiology.

[91] Khan M.A., Mujahid M. (2019). A review on recent advances in chitosan based composite for hemostatic dressings. Int. J. Biol. Macromol..

[92] Leggat P.A., Smith D.R., Kedjarune U. (2007). Surgical applications of cyanoacrylate adhesives: A review of toxicity. ANZ J. Surg..

[93] Fan P., Zeng Y., Zaldivar-Silva D., Aguero L., Wang S. (2023). Chitosan-based hemostatic hydrogels: The concept, mechanism, application, and prospects. Molecules.

[94] Kozen B.G., Kircher S.J., Henao J., Godinez F.S., Johnson A.S. (2008). An alternative hemostatic dressing: Comparison of celox, hemcon, and quikclot. Acad. Emerg. Med..

[95] Pusateri A.E., Mccarthy S.J., Gregory K.W., Harris R.A., Cardenas L., Mcmanus A.T., Goodwin C.J. (2003). Effect of a chitosan-based hemostatic dressing on blood loss and survival in a model of severe venous hemorrhage and hepatic injury in swine. J Trauma.

[96] Xia L., Wang S., Jiang Z., Chi J., Yu S., Li H., Zhang Y., Li L., Zhou C., Liu W. (2021). Hemostatic performance of chitosan-based hydrogel and its study on biodistribution and biodegradability in rats. Carbohydr. Polym..

[97] Zhao X., Wu H., Guo B., Dong R., Qiu Y., Ma P.X. (2017). Antibacterial anti-oxidant electroactive injectable hydrogel as self-healing wound dressing with hemostasis and adhesiveness for cutaneous wound healing. Biomaterials.

[98] Guo S., Ren Y., Chang R., He Y., Zhang D., Guan F., Yao M. (2022). Injectable self-healing adhesive chitosan hydrogel with antioxidative, antibacterial, and hemostatic activities for rapid hemostasis and skin wound healing. Acs Appl. Mater. Interfaces.

[99] Medzhitov R. (2008). Origin and physiological roles of inflammation. Nature.

[100] Liu Q., Huang Y., Lan Y., Zuo Q., Li C., Zhang Y., Guo R., Xue W. (2017). Acceleration of skin regeneration in full-thickness burns by incorporation of bfgf-loaded alginate microspheres into a cmcs-pva hydrogel. J. Tissue Eng. Regen. Med..

[101] Gull N., Khan S.M., Butt O.M., Islam A., Shah A., Jabeen S., Khan S.U., Khan A., Khan R.U., Butt M. (2020). Inflammation targeted chitosan-based hydrogel for controlled release of diclofenac sodium. Int. J. Biol. Macromol..

[102] Gonzalez A.C., Costa T.F., Andrade Z.A., Medrado A.R. (2016). Wound healing—A literature review. An. Brasil. Dermatol..

[103] Liu F., Wang L., Zhai X., Ji S., Ye J., Zhu Z., Teng C., Dong W., Wei W. (2023). A multi-functional double cross-linked chitosan hydrogel with tunable mechanical and antibacterial properties for skin wound dressing. Carbohydr. Polym..

[104] Larouche J., Sheoran S., Maruyama K., Martino M.M. (2018). Immune regulation of skin wound healing: Mechanisms and novel therapeutic targets. Adv. Wound Care.

[105] Chen X., Cao X., Jiang H., Che X., Xu X., Ma B., Zhang J., Huang T. (2018). Sikvav-modified chitosan hydrogel as a skinsubstitutes for wound closure in mice. Molecules.

